# Exploring the Genotype at *CSN3* Gene, Milk Composition, Coagulation and Cheese-Yield Traits of the Sardo-Modicana, an Autochthonous Cattle Breed from the Sardinia Region, Italy

**DOI:** 10.3390/ani10111995

**Published:** 2020-10-30

**Authors:** Michele Pazzola, Giuseppe Massimo Vacca, Antonia Noce, Marta Porcedda, Maria Onnis, Nicola Manca, Maria Luisa Dettori

**Affiliations:** 1 Department of Veterinary Medicine, University of Sassari, via Vienna 2, 07100 Sassari, Italy; gmvacca@uniss.it (G.M.V.); noce@fbn-dummerstorf.de (A.N.); marta.porcedda@hotmail.it (M.P.); monnis88@gmail.com (M.O.); ilmanca@yahoo.it (N.M.); mldettori@uniss.it (M.L.D.); 2 Leibniz-Institute for Farm Animal Biology (FBN), Wilhelm-Stahl-Allee 2, 18196 Dummerstorf, Germany

**Keywords:** milk, coagulation, local cattle breeds, extensive farming

## Abstract

**Simple Summary:**

The Sardo-Modicana is a local cattle breed from Sardinia, Italy. It originated from the crossing of Sardinian local cows with Modicana bulls from Sicily, imported in the late 19th century. In the 1950s, approximately 60,000 heads were present, but nowadays the total population has decreased to about 1800 animals. It is a multipurpose breed, and animals are farmed using extensive methods. Traditionally, cows are hand-milked and milk is destined to produce a traditional pasta filata cheese. In the literature, the information about the dairy potential of this breed is scarce. The present study evidenced the favorable genetic patterns, milk composition and coagulation traits of the Sardo-Modicana cattle breed; such information will be useful for the preservation and enhancement of the breed.

**Abstract:**

The Sardo-Modicana is a local cattle breed from Sardinia, Italy. No information about its dairy potential is available in the literature. This study investigated the genotype at the *CSN3* gene and milk traits of the Sardo-Modicana cattle breed. Fifty-four cows were sampled for DNA extraction and genotyping at the κ-casein gene locus, *CSN3*. Forty individual milk samples were analyzed for milk composition, milk coagulation properties and cheese yield (CY%). All the Sardo-Modicana cows were BB homozygotes at *CSN3*. Hence, the results were compared with the other two local Sardinian breeds. Eighty-three Sarda and 21 Sardo-Bruna cows were genotyped, and the A allele was found (at frequencies of 0.416 and 0.405, respectively). As regards milk traits, the mean protein value was 3.74 g/100 mL, and the mean casein value was 2.98 g/100 mL. Total bacterial and somatic cell counts showed excellent levels of hygiene considering the extensive farming and hand milking. In addition, milk produced by Sardo-Modicana cows was characterized by favorable values of coagulation properties and cheese yield. This information may represent a starting point for the conservation and enhancement of this breed.

## 1. Introduction

Sardinia is an insular region of Italy in the middle of the Mediterranean Sea, with an agricultural economy that is primarily based on dairy sheep farming [1]. Nevertheless, cattle farming is also present with extensive and intensive methods and the utilization of both foreign specialized and local breeds. Three local breeds are officially recognized, the Sarda, the Sardo-Bruna and the Sardo-Modicana [2], with standards and herd books managed by the Italian breeders’ association [3].

The Sardo-Modicana originated from the crossing of Sardinian local cows with Modicana bulls from Sicily, imported in the late 19th century to improve the size and aptitude for draught purposes. The crossbred cattle were soon appreciated by breeders because of its adaption to the Sardinian environment; it spread in many areas of the island and numbered around 60,000 heads in the 1950s [4]. Nowadays, the total population has significantly declined to about 1800 heads in 110 herds [5], figures that are also low in comparison to those of the Sarda (17,000 heads in 750 herds) and the Sardo-Bruna (20,000 heads in 1000 herds).

The marked decrease of draught cattle led to the transformation of ancient breeds into meat, dairy or multipurpose types, but some others became extinct [6]. Zander et al. [7] reported that 27% of European cattle breeds are considered at risk or endangered. Several strategies have been developed for the recovery of local breeds, such as linking to typical products that have a relationship with the social and cultural traditions of the production area [8]. In the European Union, Protected Designation of Origin (PDO) and Protected Geographical Indication (PGI) labels are a suitable tool to establish a close link with the production area, increase the economic growth and social well-being of those areas [9] and, finally, enhance biodiversity [8].

Sardo-Modicana cows are mainly reared in the mountain area of Montiferru, located in the Province of Oristano (Sardinia, Italy), where a typical pasta filata cheese called Casizolu is produced, normally at the familiar level, from their milk. Although Casizolu cheese has no official EU label, together with the Sardo-Modicana breed, it is listed among the Sardinian presidia of the Slow Food Foundation for Biodiversity [10]. These circumstances represent a positive starting point for further official recognition and the consequent recovery of an endangered breed. Nevertheless, information regarding the Sardo-Modicana cattle and the dairy products is completely missing in the available literature. Besides the loss of biodiversity, the concurrent absence of investigation of the genetic potential of endangered cattle populations has a negative impact on agricultural systems based on extensive farming [11].

Genetic studies on dairy ruminants are often focused on genotyping of the casein gene cluster. Caseins α_s1_, β, α_s2_ and κ are the main proteins in milk, coded by the four related genes *CSN1S1*, *CSN2*, *CSN1S2* and *CSN3* linked in a cluster of about 250 Kb, which in cattle is located in chromosome 6. For each of the four genes, several variants and their effects on milk production and quality are reported in the literature [12]. In particular, the *CSN3* locus is intensely studied because of its influence on cheesemaking properties. Indeed, κ-casein contains the cleavage site for chymosin, which starts the primary phase of rennet-induced coagulation [13]. To date, 14 allele variants have been described in cattle for the κ-casein gene; the allele B has positive effects on milk coagulation and cheese-making traits, and bulls used in artificial insemination programs are usually genotyped at *CSN3* [14,15].

Hence, the aim of the present study was to investigate the dairy potential of the Sardo-Modicana cattle breed by exploring both the κ-casein gene locus and milk traits.

## 2. Materials and Methods

### 2.1. Ethics Approval

No specific authorization from an animal ethics committee was required. Blood samples were collected by official veterinarians of the local health authorities (ASL) during control or eradication sanitary programs not linked with the present study. As regards milk samples, cows were not subjected to any invasive procedures. Animals belonged to commercial private herds and were sampled for the present study with the agreement of the farmers on a voluntary basis.

### 2.2. Farms and Animals

Fifty-four cows of Sardo-Modicana breed from five different herds were sampled for the present study. All farms were located in Sardinia and managed using the following extensive techniques: Heifers were bred for the first time at the age of 24 months, and reproduction was based on natural mating with a sex ratio of about 1/25. Animals were fed at pasture on a typical Mediterranean shrubland including natural grasses where the common plants were cork oak (*Quercus suber*), holm oak (*Quercus ilex*), mastic (*Pistacia lentiscus*), prickly juniper (*Juniperus oxycedrus*) and rock rose (*Cistus* spp.). Cows browsed without their own calves, which were penned together at the farms. After browsing, cows returned to the farms to feed their calves and to be milked one by one following the traditional technique: each individual calf was allowed to pass the fence, join its related cow and begin suckling of a quarter of the udder; after removal of the calf, the individual cow was finally hand-milked. Cows were milked once a day. Hence, calves were milk-fed by their mothers and later let free to pasture but separated from cows. Calves were normally weaned at the age of 8 months and fattened up to the age of about 18 months, which was the optimal age for slaughtering.

### 2.3. Blood Samples and Genotyping of CSN3 locus

Individual blood samples from the caudal vein were collected in K3EDTA vacuum tubes (Vacutest Kima, Azergrande, PD, Italy) to investigate genotype at *CSN3* locus. DNA was extracted using the Gentra Puregene Blood Kit (QIAGEN, Venlo, The Netherlands) according to the manufacturer’s instructions. The DNA samples were amplified using a specific PCR protocol for the bovine κ-casein gene, as reported by Da-Xi et al. [16]. Nucleotide positions in the present study refer to the sequence of the bovine κ-casein gene published with accession number GenBank X14908.1. The two primers were as follows: forward primer κ-bovF, 5′- CAG GTC ACC CAC ACC ACA ATT TATC -3′; reverse primer κ-bovR, 5′- TAA TTA GCC TTC TCT GT -3′. The amplification reaction was carried out in a final volume of 25 μL under the following conditions: 1 μL of genomic DNA; 2.5 μL PCR buffer (NZYTech, Lisboa, Portugal); 0.75 μL of MgCl_2_ (NZYTech, Lisboa, Portugal); 2 μL dNTPs; 0.5 μL of each primer; 0.1 μL of NZYtaq (NZYTech, Lisboa, Portugal) DNA polymerase (NZYTech, Lisboa, Portugal) and H_2_O up to the final volume. The amplification programs were performed on Mastercycler EP Gradient S (Eppendorf, Darmstadt, Germany) according to the following scheme: initial denaturation at 95 °C for 5 min; 30 cycles at 95 °C for 1 min (denaturing), 57 °C for 1 min (annealing) and 72 °C for 1 min (extension); and a final extension at 72 °C for 7 min. The PCR amplified a fragment of 379 bp, located between nucleotides 5189 and 5568 of the examined sequence, corresponding to exon 4 of κ-casein. The PCR products (8 μL of amplified DNA colored with 1 μL of blue bromophenol solution) were examined by electrophoresis on agarose gel at a concentration of 2%, using a 1 kb marker (100 bp to 1 kb log scale). The electrophoretic run lasted 45 min at 80 V, followed by staining with ethidium bromide (0.5 mg/mL) for 10 min and subsequent reading with the UV transilluminator to visualize the bands of the amplified DNA fragment. Each amplification product was subjected to polymorphism analysis for the identification of the gene variant of κ-casein using the restriction fragment length polymorphism (RFLP) technique and the restriction enzyme *Hinf* I according to the protocol by Da-Xi et al. [16]. The reaction was carried out at 37 °C for 3 h in a thermostated bath, in a final volume of 25 μL: 3.5 μL of buffer enzyme, 0.3 μL of BSA (Bovine Serum Albumin, Thermo Fisher Scientific, Waltham, MA, USA), 2 μL of *Hinf* I, 15 μL of the PCR product and H_2_O up to the final volume.

### 2.4. Milk Samples and Analyses of Milk Traits

Forty Sardo-Modicana cows out of the 54 sampled for genotyping were used to investigate milk traits. Milk samples were not collected for some of the 54 genotyped cows, because in two out of the five farms cows were not milked. Cows were primiparous (average age 3 years) and multiparous (from 4 to 14 years) and between the 3rd and 5th month of lactation. These cows were from three different farms located in the Montiferru area (Province of Oristano, Sardinia, Italy) and managed using extensive techniques as described above.

Individual milk samples were collected in 100-mL disposable sterile plastic containers. Given that an indefinite volume of milk was suckled by the calf, it was not possible to measure daily milk yield. Milk samples were stored at 4 °C and analyzed within 24 h of collection for milk composition, milk coagulation properties (MCPs) and cheese-yield (CY%). Fat, protein, casein and lactose content and pH were measured by a MilkoScan FT6000 (Foss Electric A/S, Hillerød, Denmark), according to the ISO-IDF standard [17]. Total bacterial count (TBC) was measured using a BactoScan FC150 instrument (Foss Electric) according to ISO-IDF standard [18]. Somatic cell count (SCC) was measured using a Fossomatic 5000 equipment (Foss Electric) according to the ISO-IDF method [19]. In order to normalize the distributions before statistical analysis, TBC was transformed into log bacterial count (LBC = log10 (total bacterial count/1000)) and SCC into somatic cell score (SCS = log2 (SCC × 10^−5^) + 3). The MCP traits and CY% were measured using the Formagraph instrument (Foss Italia, Padova, Italy) and the method described by McMahon and Brown [20] and later modified into the 9-MilCA protocol by Cipolat-Gotet et al. [21]. For each individual milk sample, 9 mL was heated to 35 °C and mixed with 180 μL of the rennet solution 1.2% (wt/vol) obtained by the dilution of Hansen Naturen Plus 215 (Pacovis Amrein AG, Bern, Switzerland; 80 ± 5% chymosin and 20 ± 5% pepsin; 215 international milk clotting units (IMCU)/mL) in distilled water (final milk clotting units: 0.0513 IMCU/mL of milk). The analysis lasted 30 min after rennet addition to achieve RCT (rennet coagulation time in min), k_20_ (curd firming time in min) and a_30_ (curd firmness 30 min after rennet addition, in mm). The fresh curd obtained from the Formagraph instrument was later submitted to cutting, pressing and draining, as reported in the 9-MilCA protocol [21], to calculate the CY% as the ratio of the weight of the fresh curd to the weight of the 9 mL of milk mixed with rennet solution.

In order to investigate the phenotypic effects, data regarding milk traits were submitted to a general linear procedure based on a model with the fixed effects of the farm (three levels: farm A (8 cows) vs. farm B (19 cows) vs. farm C (13 cows)), parity (two levels: primiparous (7 cows) vs. multiparous, (33 cows)) and the stage of lactation (two levels: <90 days in milking (DIM) (20 cows) vs. >90 DIM (20 cows)). The statistical analysis was performed using the Minitab software (version 13.32, Minitab Inc. 2000, State College, PA, USA), the effects of the model were declared significant at *p* < 0.05 and the Bonferroni method was used for multiple comparisons between the levels.

## 3. Results and Discussion

### 3.1. Genotyping of CSN3 Locus

**Figure 1:**
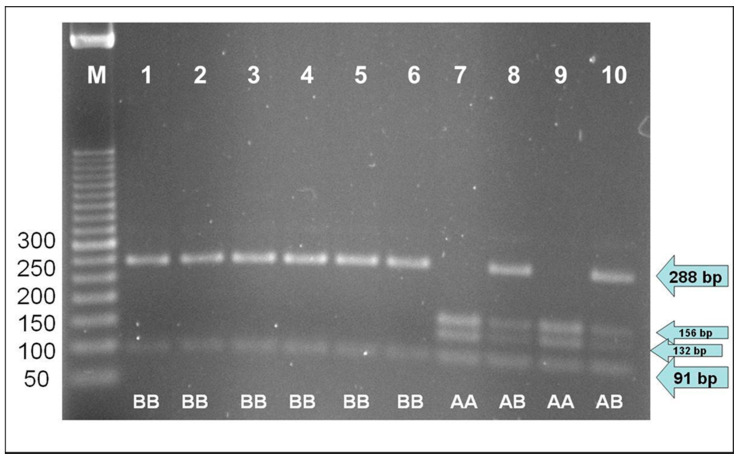
PCR-RFLP genotyping at bovine *CSN3*. Lane M: marker; lanes 1 to 6: genotype BB, Sardo-Modicana cattle breed; lanes 7 and 8: genotypes AA and AB, Sarda cattle breed; lanes 9 and 10: genotypes AA and AB, Sardo-Bruna cattle breed.

The genotyping analyses allowed investigating the allele variants at the κ-casein gene. As expected, the size of the PCR-amplified fragment was 379 bp for all animals. Figure 1 shows the image of the electrophoretic analysis of DNA samples digested by the restriction enzyme *Hinf* I. The replacement of adenine with cytosine at the enzyme recognition site results in the synthesis of the amino acid threonine in allele B, compared to the amino acid isoleucine for A. It is hypothesized that in milk samples produced by animals carrying the allele B, the isoform of κ-casein cleaved by the rennet enzyme during the first phase of milk coagulation promotes better coagulation processes [13].

Table 1 shows the allele frequencies at the *CSN3* locus. All the Sardo-Modicana cows analyzed for the present study were monomorphic, with allele B as the only one found. Even if monomorphic data are not suitable to calculate Hardy–Weinberg equilibrium, the extreme frequency of allele B evidenced some events that have favored this allele in the sampled population, e.g., due to the extreme reduction of the population throughout the last century. In order to compare this singular result with the other two local Sardinian breeds, further blood samples from 83 Sarda cows in five herds and 21 Sardo-Bruna cows in six herds were also collected, with DNA being extracted and genotyped using the same methods as mentioned above. These farms were located in Sardinia and managed using extensive techniques already described for the Sardo-Modicana. In Sarda and Sardo-Bruna, the *CSN3* A was found as the minor allele, at frequencies of 0.416 and 0.405, respectively (Figure 1 and Table 1).

The monomorphism of the *CSN3* B allele evidenced in the Sardo-Modicana cows of the present study is different than data recorded from the original breed from which the Sardo-Modicana is derived. Indeed, Marletta et al. [22] have found a frequency of the B allele at 0.786 for the Modicana in Sicily. Since κ-casein plays a key role in cheese making, influencing the yield and the characteristics of the curd, it seems likely that the genetic polymorphism of this locus is not completely free from selective pressure. In fact, Ward et al. [23] provided evidence that, within the family Bovidae, *CSN3* alleles’ frequencies are related to a positive selective pressure which has caused a high level of polymorphism in many of the proteins but not in κ-casein.

The comparison with other species and breeds evidences the peculiarity of the Sardo-Modicana cattle. Comin et al. [24] reported an extreme prevalence of *CSN3* genotypes AA and AB in the Italian Friesian cattle. These results are expected because of the strong influence exerted by the selective goal of Friesian cattle breeders, which is mainly used to produce drinking milk. Indeed, breeders prefer bulls in selective plans to increase milk yield, which are often carriers of the AA or AB genotype and rarely carriers of the BB genotype. A similar situation was reported by Maletić et al. [25] in a population of Red and White Holstein sampled in Serbia, with a very high frequency of the genotypes AA (44%) and AB (50%), rather than the homozygous BB (5.6%). The selective objectives pursued by breeders of Friesian cows in Europe, and the consequent genetic patterns, are different from those in the Middle East. Doosti et al. [26] recorded a higher frequency of the allele B (0.41) in Holstein Friesian cows in Iran. Similarly, Da-Xi et al. [16] reported *CSN3* B allele frequencies of 0.31 and 0.88 in Holstein and Jersey cows, respectively, as well as reporting the sole B allele in the local water buffalo.

### 3.2. Milk and Cheese-Yield Traits

Results of statistical analysis and least square means of milk traits according to the phenotypic effects are reported in Table 2. Data regarding daily milk yield is missing because of the milking technique. Indeed, none of the Sardo-Modicana cows of the present study were milked to obtain the complete milk yield. The udder was not completely milked because the calf suckled a limited and nonconstant volume of milk, and milking was always performed after the calf had suckled a portion of the potential total milk yield. Fat content, characterized by a large interval, was similarly affected by the milking technique and the consequent difficulties in obtaining an appropriate milk sampling. This method is traditionally used in nonspecialized dairy cows to stimulate the release of oxytocin, contraction of myoepithelial cells, squeezing of mammary alveoli and finally milk ejection [27]. Consequently, we hypothesize an altered transfer of milk fat from the alveoli to the cistern because fat is stratified in the upper side of the gland cistern and in the alveoli [28]. Hence, the reliability of the actual content of milk fat in the present study and the comparison with other studies performed by standard sampling and analysis methods was influenced by the milking method when compared with intensive automated milking. Nevertheless, the average fat content values recorded by two different studies in the original breed, the Modicana from Sicily, were 3.58% and 4.43% [29,30]. Regarding the comparison of the other milk traits between the two breeds, mean values from Sardo-Modicana cows were similar to Sicilian Modicana [29] both for protein (3.74 vs. 3.59 g/100 mL) and casein content (2.98 vs. 2.78 g/100 mL). As for the protein content, the mean value in Sardo-Modicana is favorable if compared with cosmopolitan and local breeds farmed in Italy (Brown Swiss, Grigia Alpina, Rendena, Simmental and Italian Friesian) [31]. Finally, lactose and pH were in accordance with general data recorded for cow milk [32].

Most of the individual milk samples collected in the present study showed excellent levels of milk hygiene considering the extensive farming and the milking method. Thirty-three samples out of 40 (data not shown in tables), had TBC lower than 100,000 cells/mL, which is the maximum limit for raw bulk milk in European Union [33] and United States legislation [34]; 35 out of 40 had SCC lower than 750,000 cells/mL, which is the maximum limit for raw bulk milk in the United States [34]; and 27 out of 40 had SCC even lower than the limit for “high quality” pasteurized milk set by the Italian national legislation for bulk milk (300,000 somatic cells/mL) [35]. As regards cellular parameters, mean value (± standard deviation) for TBC were 45 (±156) × 1000 cells/mL and for SCC were 370 (±547) × 1000 cells/mL (data not shown in tables).

Despite the differences in rennet solutions between the two protocols, MCPs recorded in the present study were overall not as good as those of the original Modicana from Sicily [30]. The mean value of rennet coagulation time, 17.28 min, was intermediate among the values of the five Italian cattle breeds reported by De Marchi et al. [31]. The comparison with the same study by De Marchi et al. [31] indicates that k_20_ of the Sardo-Modicana, 4.60 min, is the shortest among those breeds and the a_30_ of the Sardo-Modicana, 33.88 mm, is the highest among those breeds. Moreover, the mean value of CY% recorded for the Sardo-Modicana, 17.29%, is satisfactory if compared with other Italian breeds, in which the average CY% is 15.18 [21]. Hence, milk produced by Sardo-Modicana cows is characterized by suitable values of coagulation properties and cheese yield.

Among the effects considered in the statistical analysis (Table 2), fat, lactose and pH were influenced by the farm. This finding may be due to different aspects at the farms (e.g., management, environment, pastures, nutrition) the difference found for fat is mainly attributable to the milking procedures, the different milking operators among the farms and the different volume of milk suckled by the calves. The effect of parity did not affect any milk traits, and this finding is not in accordance with the literature since parity order is normally associated with changes in milk composition [36]. As regards the stage of lactation, cows with >90 DIM, compared to those with <90 DIM, showed the highest values of protein and CY%.

## 4. Conclusions

The dairy potential of the Sardo-Modicana, a local cattle breed from Italy with a small population size, was explored for the first time. Genetic analysis, revealing the sole presence of animals with the homozygous BB genotype at the *CSN3* gene, showed a valuable genetic profile for cheese-making purposes, since the majority of milk production is used for the cheese-making and the Sardo-Modicana is perfectly adapted to the territory. Moreover, the composition and coagulation traits of milk and the predicted cheese yield are favorable if compared with those of other breeds. This information is useful as it improves the knowledge on a local breed reared with particular, extensive and traditional production systems. Hence, this breed should not be considered under the criteria of commercial and specialized breeds; rather, to provide novel data for the preservation and enhancement of a small-sized population, it should be used as a breed-specific case report and finally compared with other different local breeds and production systems worldwide. Even if further investigations are needed, with a higher number of sampled animals and herds, the information contained in this study may represent a starting point for the characterization of the breed and its dairy products and the consequent conservation of biodiversity.

## Figures and Tables

**Table 1:** Results of genotyping at *CSN3* locus in Sardinian local cattle breeds (n = 158).

Breed	n	%Gen	ObsHet	PredHet	HWpval	MAF	Alleles
Sardo-Modicana	54	100	0.0	0.0	-	0.0	B
Sarda	83	100	0.518	0.486	0.545	0.416	(A):B
Sardo-Bruna	21	100	0.429	0.481	0.612	0.405	(A):B

%Gen: percentage of genotyped samples; ObsHet: observed heterozygosity; PredHet: predicted heterozygosity; HWpval: Hardy–Weinberg test *p*-value; MAF: minor allele frequency (minor allele in brackets).

**Table 2:** Least square means of milk traits from Sardo-Modicana cows (n = 40) and significance according to the phenotypic effects.

Effects	Farm				Parity ^1^			Days in Milking		
Levels	A	B	C	*p*-value	P	M	*p*-value	<90	>90	*p*-value
(n)	(8)	(19)	(13)		(7)	(33)		(20)	(20)	
Milk traits ^2^										
Fat (g/100 mL)	2.40 ^a^	2.89 ^ab^	3.02 ^b^	0.001	2.80	2.87	0.137	2.71	2.72	0.921
Protein (g/100 mL)	3.69	3.85	3.84	0.305	3.92	3.66	0.051	3.66 ^a^	4.00 ^b^	0.001
Casein (g/100 mL)	2.90	3.08	3.08	0.130	3.13	2.90	0.052	2.92 ^a^	3.22 ^b^	0.001
Lactose (g/100 mL)	5.21 ^ab^	5.20 ^a^	5.39 ^b^	0.014	5.19	5.22	0.831	5.16	5.26	0.155
pH	6.68 ^ab^	6.67 ^a^	6.76 ^b^	0.001	6.68	6.74	0.092	6.72	6.70	0.263
LBC	3.88	3.93	3.76	0.509	3.76	3.93	0.710	3.95	3.73	0.390
SSC	7.00	6.89	6.67	0.486	6.44	7.26	0.053	6.93	6.78	0.503
RCT (min)	17.40	16.96	18.80	0.160	18.40	17.36	0.634	15.84	17.64	0.896
k_20_ (min)	4.55	4.75	4.30	0.221	4.24	4.71	0.539	4.54	4.42	0.741
a_30_ (mm)	34.66	34.97	29.95	0.064	30.66	33.56	0.570	31.27	32.95	0.570
CY%	16.85	17.10	16.37	0.191	16.23	17.26	0.274	15.84 ^a^	17.64 ^b^	0.002

^1^ P: primiparous; M: multiparous. ^2^ LBC = log10 (total bacterial count/1000); SCS = log2 (SCC × 10^−5^) + 3; RCT: rennet coagulation time; k_20_: curd firming time); a_30_: curd firmness 30 min after rennet addition; CY%: cheese yield as the ratio between the weights of the fresh curd and milk. ^a,b^ Means with different superscript letters for each effect differ significantly in level comparison at the indicated *p*-value.
